# Remote sensing and geographic information systems: charting Sin Nombre virus infections in deer mice.

**DOI:** 10.3201/eid0603.000304

**Published:** 2000

**Authors:** J D Boone, K C McGwire, E W Otteson, R S DeBaca, E A Kuhn, P Villard, P F Brussard, S C St Jeor

**Affiliations:** University of Nevada, Reno, Nevada 89557, USA. boone@scsr.nevada.edu

## Abstract

We tested environmental data from remote sensing and geographic information system maps as indicators of Sin Nombre virus (SNV) infections in deer mouse (Peromyscus maniculatus) populations in the Walker River Basin, Nevada and California. We determined by serologic testing the presence of SNV infections in deer mice from 144 field sites. We used remote sensing and geographic information systems data to characterize the vegetation type and density, elevation, slope, and hydrologic features of each site. The data retroactively predicted infection status of deer mice with up to 80% accuracy. If models of SNV temporal dynamics can be integrated with baseline spatial models, human risk for infection may be assessed with reasonable accuracy.


### Remote Sensing and Geographic Information Systems: Charting Sin Nombre Virus Infections in Deer Mice

John D. Boone,* Kenneth C. McGwire,† Elmer W. Otteson,* Robert S. DeBaca,† Edward A. Kuhn,* Pascal Villard,* Peter F. Brussard,* and Stephen C. St. Jeor*
*University of Nevada, Reno, Nevada, USA; and †Desert Research Institute, Biological Sciences Center, Reno, Nevada, USA

We tested environmental data from remote sensing and geographic information system maps as indicators of Sin Nombre virus (SNV) infections in deer mouse (***Peromyscus maniculatus***) populations in the Walker River Basin, Nevada and California. We determined by serologic testing the presence of SNV infections in deer mice from 144 field sites. We used remote sensing and geographic information systems data to characterize the vegetation type and density, elevation, slope, and hydrologic features of each site. The data retroactively predicted infection status of deer mice with up to 80% accuracy. If models of SNV temporal dynamics can be integrated with baseline spatial models, human risk for infection may be assessed with reasonable accuracy.

Remote sensing (RS) and geographic information systems (GIS) are map-based tools that can be used to study the distribution, dynamics, and environmental correlates of diseases (1,2). RS is gathering digital images of the earth's surface from airborne or satellite platforms and transforming them into maps. GIS is a data management system that organizes and displays digital map data from RS or other sources and facilitates the analysis of relationships between mapped features. Statistical relationships often exist between mapped features and diseases in natural host or human populations (1). Examples include malaria in southern Mexico and in Asia (3,4), Rift Valley fever in Kenya (5), Lyme disease in Illinois (6), African trypanosomiasis (7), and schistosomiasis in both humans (8) and livestock in the southeastern United States (9). RS and GIS may also permit assessment of human risk from pathogens such as Sin Nombre virus (SNV; family Bunyaviridae), the agent primarily associated with hantavirus pulmonary syndrome (HPS) in North America (10,11). RS and GIS are most useful if disease dynamics and distributions are clearly related to mapped environmental variables. For example, if a disease is associated with certain vegetation types or physical characteristics (elevation, average precipitation), RS and GIS could identify regions where risk is relatively high.

We examined whether RS and GIS data were useful indicators of the spatial pattern of SNV infections in populations of the primary rodent host, the deer mouse (***Peromyscus maniculatus***) (12-15). Our approach involved determining the infection status of rodents at 144 field sites, collecting RS and GIS data for each site, testing for statistical relationships between these data and infection, using the statistical relationships to retroactively classify infection status of rodents at these sites, and using the classifications to estimate prediction accuracy. Predictions derived from RS and GIS data could identify the ecologic settings where human exposure to SNV is most likely to occur.

### SNV and its Host

Since the first recognized outbreak of HPS in the southwestern United States in 1993, approximately 240 cases have occurred, with a

Address for correspondence: John D. Boone, Dept. of Microbiology/320, University of Nevada, Reno, NV 89557, USA; fax: 775-784-1620; e-mail: boone@scsr.nevada.edu.

death rate of approximately 40% (J. Mills, pers. comm.) (16). Information about SNV host-virus-environment relationships is limited (16,17). No simple relationships have been found between host density and antibody seroprevalence (16-18), but more complex nonlinear relationships appear to exist (17). SNV infections also appear to be less frequent in relatively high- or low-elevation habitats (16,17).

### Study Design

### Types of Data

RS data are commonly used to generate maps of vegetation types. Vegetation types can be useful indicators of environmental characteristics, including moisture, soil type, and elevation. However, transforming RS images into vegetation maps can be subjective and imprecise (19,20); therefore, we supplemented our vegetation maps with other RS/GIS data, including elevation, slope, vegetation density, and hydrology.

We sampled rodents over four field seasons (June to October during 1995 to 1998). However, in 1997, population densities of deer mice in our study area averaged approximately 25% of 1995-96 and 1998 levels (unpub. data, Boone et al.). Most of the 47 sites sampled in 1997 had three or fewer deer mice, and 14 sites had none. Simple t-tests (SAS ver. 6.10) showed that the mean number of animals per site was statistically equivalent in 1995, 1996, and 1998 (11.1, 10.3, and 8.9 animals per site, respectively; $p > 0.10$ for all comparisons), but differed significantly in 1997 from all other years (2.4 animals per site; $p < 0.0001$ for all comparisons). In 1997, antibody-positive animals were significantly less likely to be positive by reverse transcription-polymerase chain reaction (RT-PCR) for viral RNA in the blood than in any other year, suggesting unusual infection patterns (25% were PCR positive in 1997 and >50% to 70% in other years; chi-square test, $p < 0.002$ for all comparisons of 1997 to other years; $p > 0.20$ for all comparisons of years excluding 1997). On the basis of these tests, we pooled data from 1995, 1996, and 1998 and excluded 1997 data from all analyses because host density and infection dynamics appeared atypical and likely to obscure the baseline spatial infection patterns we sought to identify (21).

### Infection Status of Sites

Presence of SNV infections is commonly inferred by determining antibody seroprevalence in a host population (14,16-18,21-23). However, antibody prevalence at the same site may vary considerably (<5% to >60%) over relatively brief periods of <1 year (17,18,22,23), probably because of rapid turnover of rodent populations through death, reproduction, dispersal, and migration. We focused on the presence or absence of SNV infections inferred from antibody data, a more stable measure than antibody prevalence. However, determining infection status is complicated by several factors: animals may remain antibody-positive well after the transmissible phase of an infection (17); noninfectious but antibody-positive deer mice may migrate to a site where no active SNV infection is present; and detectable antibody response requires at least 1 to 2 weeks to develop in newly infected animals (17).

Because of these uncertainties, we used two criteria to demonstrate the effect of classification on analytical outcome. “Status 1” classified sites with one or more antibody-positive animals as positive (active infection present). This criterion may have falsely assigned positive status to some sites where no active, transmissible infections were present. “Status 2” required two or more antibody-positive deer mice or an overall antibody seroprevalence of at least 10% for a site to be classified positive. This criterion may have falsely assigned negative (active infection absent) status to some sites that had a single infectious animal.

### Site Selection

Our study area was the Walker River Basin, a 10,200-$km^2$ region in western Nevada and east-central California northeast of Yosemite National Park (Figure 1). At least nine cases of HPS have occurred in the area since 1993. Major vegetation types in the river basin along an increasing elevational gradient (1,200 m to 3,760 m) are salt desert scrub, sagebrush-grass scrub, piñon-juniper woodland, coniferous forest, montane shrubland, and alpine tundra, with riparian habitat and meadows at a wide range of elevations (24).

We compiled a GIS database for the study area, including a second-generation map of vegetation types (Figure 1) (25). The vegetation

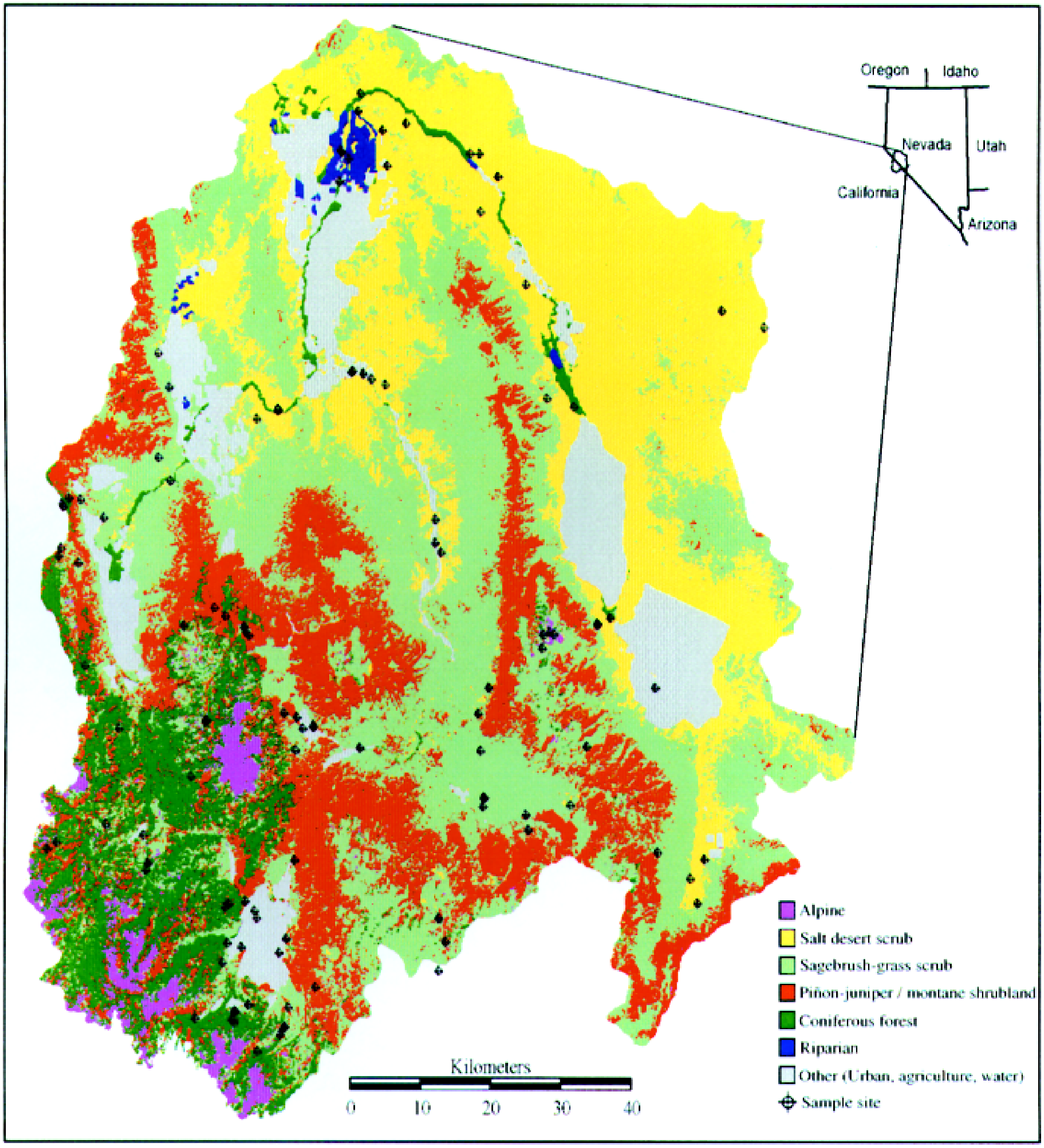

Figure 1. Location of Walker River Basin (17) and its eight major vegetation types, as well as developed areas. Piñon-juniper woodland and montane shrubland tend to be highly interspersed and were combined for visual clarity. Because meadows occurred in very small patches, they could not be represented on this map. Map generated at Utah State University as part of the GAP conservation mapping project.

map, which was generated from Landsat Thematic Mapper images and digital elevation data, had a 100-hectare mapping unit. We aggregated the 36 vegetation subtypes on the GAP map into the eight general vegetation types described above. To estimate vegetation density, we used the normalized difference vegetation index (NDVI), a transformation of near infrared (TM band 4) and red wavelengths (TM band 3) correlated with the amount and productivity, or rate of plant growth, of vegetation (5,26,27). The standard deviation of NDVI within a local area was calculated to estimate the uniformity of vegetation density at each field site. Elevation and slope (i.e., steepness) data were derived from the 2-arcsecond digital elevation model of the U.S. Geological Survey. Because riparian zones could influence rodent population densities and facilitate rodent dispersal across arid regions, we calculated proximity to streams and bodies of water on the U.S. Geological Survey's 1:100,000-scale digital line graph datasets.

In 1995, we sampled rodents at 42 sites before the GAP map became available. These sites were selected as representative of the five most common vegetation types in the Walker River Basin. In 1996 and 1998, full GIS datasets and the GAP map were used to distribute 102 new field sampling sites systematically across the widest possible range of environmental conditions. We categorized each GIS variable according to its relevance to each of the eight vegetation types. For example, 'distance to streams' was a meaningful distinction within salt desert but not within riparian habitat; elevation varied substantially within sagebrush scrub but not within alpine tundra. For each vegetation type, the relevant variables were divided into high and low ranges. The resulting binary classes for each variable were then intersected in GIS to produce distinct environmental "combinations," or strata, for each vegetation type (Figure 2). Randomly located sample sites were selected within each stratum so that they were within 0.5 km of a passable road and at least 1 km from any other sample site (Figures 1,3). The number of replicates within each stratum (including 1995 sites, which were included retroactively) was proportional to its spatial extent, with a minimum sample size of two. This GIS-based stratification is a more objective and randomized variation of the gradsect sampling method (28, 29).

All samples were collected from early June to early October to minimize seasonal effects on host density and antibody prevalence (17). Seasonal influences were minimized by sampling the replicate sites within each environmental stratum at different times throughout the field season.

### Field and Laboratory Procedures

Deer mice were live-trapped at all field sites according to a fixed protocol (17). Each site had 48 live-traps in place for 3 days. A blood sample was collected from each deer mouse by retroorbital puncture with a heparinized capillary tube or Pasteur pipette. Blood samples were placed on dry ice and returned to the laboratory for enzyme-linked immunosorbent assay testing for immunoglobulin G antibody to SNV, which indicates current or past infections (14). Relative population density was estimated by counting the number of animals captured during a trapping session.

### Analytical Methods

Of the 144 sites sampled in 1995, 1996, and 1998, 25 were excluded from analysis because no deer mice were captured. Status 1 classified 38 of the remaining 119 sites as negative and 81 as positive. Status 2 classified 70 sites as negative and 49 as positive (i.e., 32 sites had differing infection status under the two criteria). We tested (by chi-square, SAS ver. 6.10, PROC FREQ) for differences among the proportion of positive sites for each vegetation type. Then, with a canonical linear discriminant function analysis [DFA] [SAS ver. 6.10, PROC DISCRIM], we examined relationships between infection status and the alternate set of RS and GIS variables with slope, elevation, density and uniformity of vegetation, and distance from streams as indicators of SNV infection status (3,17). Prior probabilities were adjusted to reflect actual proportions of positive and negative sites.

The relationships derived from these two analyses were then used to classify sites retroactively according to their expected infection status. Because error rates were not distributed evenly among sites classified as positive and negative, we present these results separately. Classification accuracy is a general estimate of the prediction accuracy of each method if it were applied to new sites in a similar

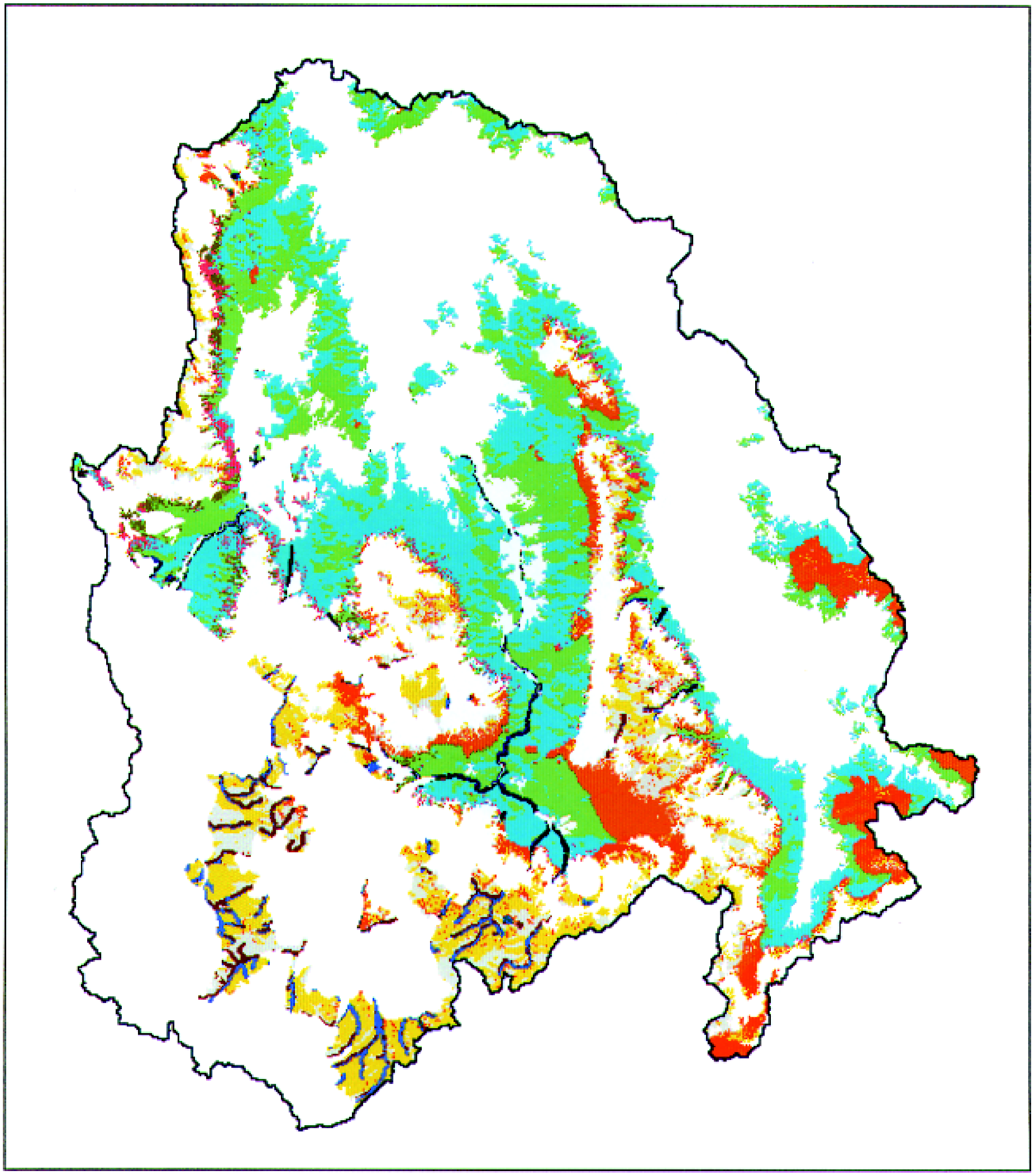

Figure 2. Environmental strata within the mapped extent of the sagebrush-grass scrub vegetation type. Each color represents a unique combination of high or low vegetation density index, standard deviation of vegetation density index, slope, elevation, and distance from stream. White areas represent the other seven vegetation types (strata not shown).

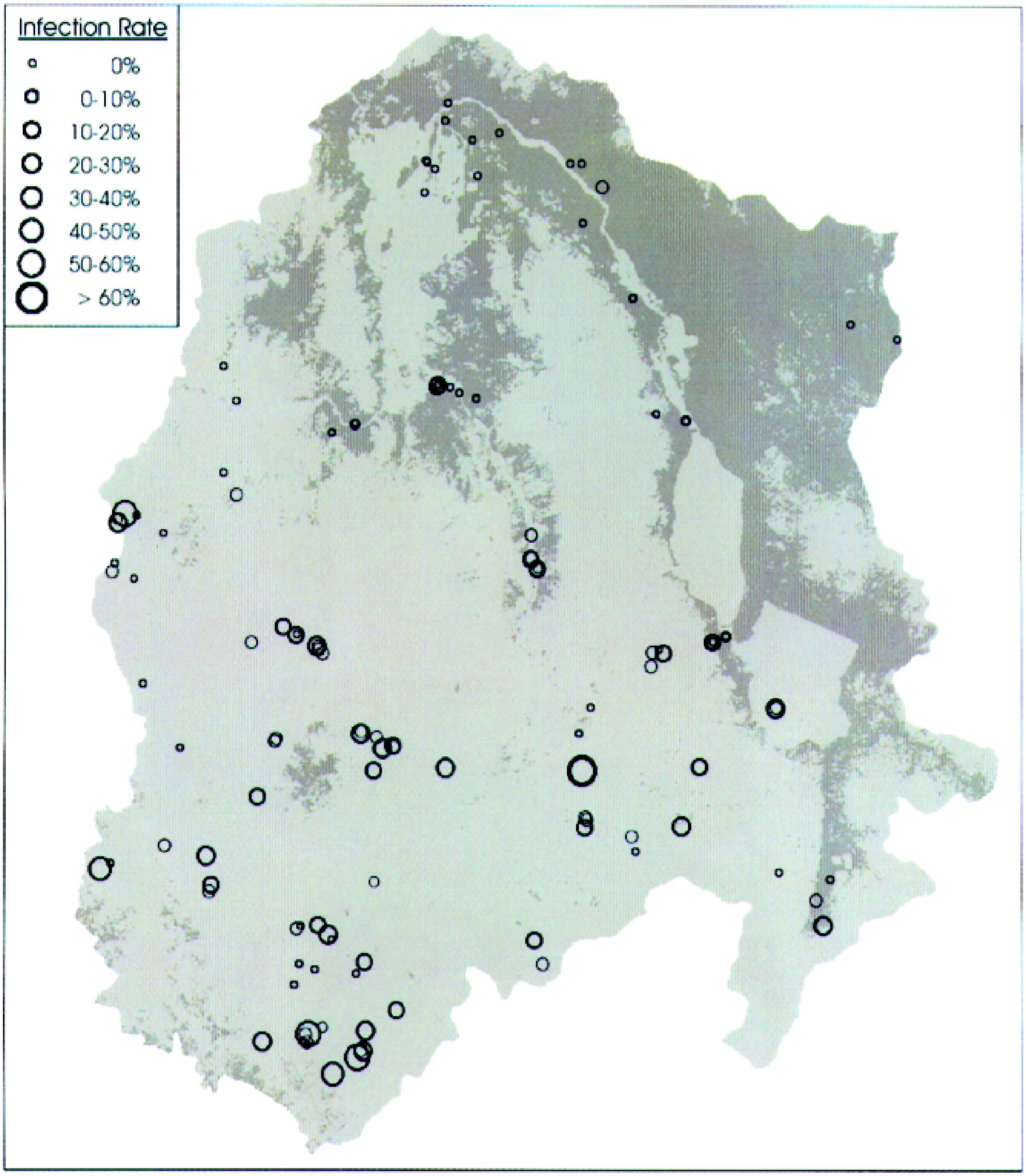

Figure 3. Seroprevalence of IgG antibody to Sin Nombre virus at sample sites in the Walker River Basin. (According to Status 1, sites with 0% prevalence were negative and all other sites were positive.)

landscape. For each prediction accuracy estimate, we calculated 95% confidence limits derived from the binomial distribution. Standard deviation for the binomial distribution is

$$\sqrt{\frac{P(1-P)}{n}}$$

where p = accuracy estimate and n = number of samples. A normal approximation of confidence limits was obtained by multiplying the standard deviation of each estimate by the t-table value associated with 95% confidence and the appropriate number of samples. These confidence intervals also allowed us to determine whether classification accuracy differed significantly between methods.

### Results

### Vegetation Types

The proportion of positive sites in salt desert scrub (34% of 29 sites by Status 1, 14% by Status 2) was significantly lower than in any other vegetation type (p = 0.05 criteria for significance). No significant differences were found among any of the other seven vegetation types, where positive sites were more common by both Status 1 (50% to 100%) and Status 2 (50% to 83%) (Figure 3). By assigning the predominant infection status to all sites within a given vegetation type, overall classification accuracies of 76% (Status 1) and 59% (Status 2) could be achieved (Table 1). The Status 1 criterion resulted in better classification accuracy (for negative sites and for all sites combined) than Status 2. For both Status 1 and Status 2, positive classification was more accurate (> 88%) than negative classification ( 50%).

DFAs for both Status 1 and Status 2 produced significant canonical correlations showing that negative sites were associated with low elevations and sparse vegetation (Table 2). These qualities most often occur in salt desert scrub (24). In contrast, positive sites were higher and generally had more dense but less uniform vegetation. Slope and distance from streams were relatively unimportant factors. For both Status 1 and Status 2, negative classification was more accurate than positive classification (Table 1). Positive classification was more accurate in Status 2 than in Status 1.

### Discussion

RS and GIS data were useful indicators of the SNV infection status of deer mice in our study area. Sites with typical salt desert scrub characteristics were less likely to have infected mice than other sites. If the 25 sites where no deer mice were captured (primarily salt desert

Table 1. Classification and prediction accuracies for Sin Nombre virus infection status, by vegetation type method and canonical discriminant function analysis

| Site in Walker River Basin | % classified negative | % classified positive | % overall accuracy |
|---|---|---|---|
| Vegetation type Status 1 | 50 ± 12[a] | 88 ± 8 | 76 ± 10 |
| Vegetation type Status 2 | 36 ± 9 | 92 ± 6 | 59 ± 12 |
| DFA[b] Status 1 | 84 ± 9 | 69 ± 11 | 74 ± 10 |
| DFA Status 2 | 84 ± 9 | 80 ± 10 | 82 ± 9 |

[a]The error terms following each estimate are 95% confidence intervals derived from the binomial distribution.
[b]DFA = discriminant function analysis.

Table 2. Sin Nombre virus infection status of sites in the Walker River Basin: canonical discriminant function analyses for Status 1 and Status 2

| | Canonical correlation[a] (p-value) | Canonical loadings[b] | | | | |
|---|---|---|---|---|---|---|
| | | Elevation | NDVI[c] | NDVI Std[d] | Slope | Streams[e] |
| Status 1 | 0.41 (0.0003) | 0.77 | 0.52 | 0.69 | 0.06 | 0.04 |
| Status 2 | 0.53 (0.0001) | 0.79 | 0.46 | 0.68 | 0.09 | 0.05 |

[a]The canonical correlation and its significance level define the overall association between infection status and the indicator variables.
[b]Canonical loadings for each indicator variable indicate their relative importance in producing a significant overall canonical correlation.
[c]NDVI = vegetation density.
[d]NDVI Std. = uniformity of vegetation density.
[e]Streams = distance to nearest mapped stream or body of water.

scrub sites) had been incorporated into our analyses as negative sites, this relationship would have been more pronounced. The relationship may be explained by the level of connectivity (i.e., biological interchange) among host populations. Salt desert scrub or similar arid habitats in the western United States are frequently dominated by heteromyid rodents (kangaroo rats, pocket mice) rather than by deer mice and other potential hosts for SNV. Although deer mice were found in salt desert scrub in the Walker River Basin and were sometimes locally abundant, their overall population density was somewhat lower than in other vegetation types, and they were more likely to be locally absent (17). We suspect that SNV infections are less likely in deer mouse populations that inhabit such regions because of their relative isolation from neighboring populations (30,31). Such fragmentation of host populations may reduce the rate of disease propagation across space and the frequency of infection recurrences within local sites. This hypothesis is supported by the clustering of negative sites in landscapes dominated by salt desert scrub (Figure 3), despite the fact that some of these sites had relatively dense deer mouse populations.

### Spatial Versus Temporal Disease Patterns

Because the RS and GIS maps summarize relatively fixed spatial properties of the environment, we focused on investigating the corresponding spatial patterns of SNV infections. SNV infections also exhibit temporal dynamics (13,16-18,22,23) superimposed on the baseline spatial pattern. However, a robust temporal study would require many years of replicated, longitudinal field data, as well as real-time RS data describing temporally variable environmental characteristics (such as climatic variables) for the corresponding period. We did not incorporate weather or climate data into GIS because weather monitoring stations are widely scattered throughout most of the study area, preventing meaningful extrapolations to most of the field sites.

### Sampling Design

Because characterizing large-scale spatial disease patterns requires a large sample size, we maximized the number of sites sampled rather than visiting fewer sites on multiple occasions. This cross-sectional approach captured substantial ecologic diversity and provided statistical replicates of sites with similar characteristics. The disadvantage of the approach was a degree of uncertainty in determining the actual infection status at each site. However, when generalization of results is an important goal, a large, replicated, and diverse dataset that has a modest degree of measurement error is statistically preferable to a smaller, more precisely measured but poorly replicated dataset (32).

### Comparison of Methods (Table 1)

The vegetation type approach was based on possible relationships between infection status and a preexisting vegetation classification that might or might not be relevant to deer mice and SNV infections. DFA, in contrast, generated a linear function that best distinguished the properties of positive and negative sites. Our results suggest that DFA yields a better balance between classification accuracies for positive and negative status (especially for Status 2).

The vegetation type method could not classify negative status as effectively as the DFA, and balance between error rates for positive and negative classifications was poor. This could be a result of using predefined vegetation types (rather than making environmental distinctions from actual infection patterns) or inaccuracies in identifying and mapping vegetation types. Site visits suggested that the DFA identified sites with pronounced salt desert features more effectively than the vegetation map. The substantial environmental variability within the mapped extent of salt desert scrub was easily captured by the set of RS and GIS variables but was analytically "invisible" to our aggregated GAP map. Some variability might have been captured by the GAP map's 36 original vegetation subclasses, but using all these in our analysis would have presented serious statistical problems.

Other analytical approaches are possible that were not presented here. For example, decision tree analysis (33,34) offers advantages if nonlinear relationships exist; hierarchical information on the effects of each predictor variable is desired; or ease of interpretation is important (29).

### Classification and Prediction Accuracy

Classification accuracy varied significantly between the Status 1 and Status 2 criteria (Table 1), with Status 2 giving better classification balance for DFA and Status 1 producing better results for the vegetation type analysis. Unfortunately, the biological significance of these analytical differences is difficult to determine. However, the infection status of 73% of the sites was classified similarly by the two criteria. The remaining 32 sites of ambiguous infection status might represent regions where infection status changes with relatively high frequency. If so, this produces an intrinsic limitation in the capabilities of the methods we present. The choice of technique might be based on the relative risks and costs of false-negative versus false-positive predictions.

Both methods may have occasionally been unable to detect positive sites because of failure to capture positive deer mice. The likelihood of this error would be proportional to the number of resident animals not captured at a site. Our longitudinal data (17; unpub. data, Boone et al.) suggest that the 3-day sampling sessions captured most of the animals present at a site. During four 7-day trapping sessions, 86% ± 9% of animals were captured during the first 3 days of trapping. Additionally, examination of 123 3-day sessions within the context of their extended longitudinal infection timelines (17; unpub. data, Boone et al.) suggested that infection status was classified with 85% (Status 1) and 81% (Status 2) accuracy.

A cross-sectional, replicated, and randomized sampling approach should capture most sites while they exhibit their most typical infection status. However, a 'background' rate of classification errors is to be expected regardless of analytical method, given the temporally dynamic nature of SNV infections (17,18,22,23). For instance, even where infection status is predominantly positive, some sites may be sampled during atypical periods when infection is temporarily absent; the reverse could also occur. Additionally, a subset of sites might frequently change their infection status and not exhibit primary infection status. Thus it might be difficult to improve upon the highest overall classification success we achieved (with DFA and Status 2 criterion), unless temporal infection dynamics are incorporated into the predictive model. Another option would be to omit sites from analysis if they fail to meet unambiguous criteria for positive or negative status; however, this might result in the loss of biological insight.

### Future Directions

We explored the ability of RS and GIS data to predict the baseline spatial patterns of SNV infections across an ecologically variable landscape. Our findings should be at least somewhat relevant to a number of other regions in the arid western United States, especially if infection dynamics are ultimately driven by host connectivity patterns. To expand these findings, we developed methods to filter environmental data to remove statistical noise and a computer simulation model to explore infection dynamics on a variety of virtual landscapes. Further work will focus on the role of landscape structure in producing spatial patterns of disease (35). For instance, deer mice in small patches of salt desert scrub within a matrix of more desirable habitat types might be more likely to be infected than mice living in large contiguous regions of salt desert scrub. Finally, it would be useful to test other types of RS and GIS data as possible indicators of SNV infections.

Further work is needed to identify possible climatic correlates of periodic outbreaks and the degree to which useful indicators of these outbreaks can be derived from RS and GIS data sources. In contrast to predictions in large-scale outbreaks, specific a priori predictions of temporal SNV infection dynamics in local sites may remain difficult. Once infections are initiated at a site (presumably by random dispersal events), changes in antibody and virus prevalence cannot be easily explained by changes in host density or environmental factors (17,18). However, it should be possible to estimate the frequency (if not the specific timing) of new infections as a function of a site's local environment. Additionally, extended longitudinal studies could identify typical infection trajectories of sites based on their environmental characteristics or demographic profiles of their host populations. When combined, these approaches should advance our ability to quantify and predict disease dynamics and human risk.

### Acknowledgments

We thank Joe Blattman for field work; Tim Wade and Kathy Bishop for GIS assistance; Jack Hayes for general advice; Joan Rowe and Jeff Riolo for laboratory assistance and advice; and Ed Volterra, Benny Romero, and George Mortenson for help with arranging field work on private and other limited-access properties.

This research was funded by NIH grants 5 RO1 AI36418-04 and 1 PO1 AI39808-01, with supporting funding from the National Aeronautics and Space Administration. JDB was supported in part by NIH Postdoctoral Fellowship F32 AI09621.

Dr. Boone is a research assistant professor, Department of Microbiology, University of Nevada, Reno. His interests include disease and animal ecology and ecologic genetics.

### References

1. Hugh-Jones M. Applications of remote sensing to the identification of the habitats of parasites and disease vectors. Parasitology Today 1989;5:244-51.
2. Glick G. The geographic analysis of cancer occurrence: past progress and future directions. In: Meade M, editor. Conceptual methodological issues in medical geography. Chapel Hill (NC): University of North Carolina Press; 1980. p. 170-93.
3. Beck L, Rodriguez M, Dister S, Rodriguez A, Rejmankova E, Ulloa A, et al. Remote sensing as a landscape epidemiologic tool to identify villages at high risk for malaria transmission. Am J Trop Med Hyg 1994;51:271-80.
4. Wood B, Beck L, Washino R, Palchick S, Sebesta P. Spectral and spatial characteristics of rice field mosquito habitat. International Journal of Remote Sensing 1991;12:621-6.
5. Linthicom K, Bailey C, Davies F, Tucker C. Detection of Rift Valley fever viral activity in Kenya by remote sensing imagery. Science 1987;235:1656-9.
6. Kitron U, Bouseman J, Jones C. Use of the ARC/INFO GIS to study the distribution of Lyme disease ticks in an Illinois county. Prev Vet Med 1991;11:243-8.
7. Rogers D, Randolph S. Mortality rates and population density of tsetse flies correlated with satellite imagery. Nature 1991;351:739-41.
8. Cross E, Perrine R, Sheffield C, Passaglia G. Predicting areas endemic for schistosomiasis using weather variables and a Landsat data base. Mil Med 1984;149:542-4.
9. Malone J, Zukowski S. Geographical models and control of cattle liver flukes in the southeastern USA. Parasitology Today 1992;8:266-70.
10. Childs JE, Rollin PE. Emergence of hantavirus disease in the USA and Europe. Current Opinion in Infectious Diseases 1994;7:220-4.
11. Nichol ST, Spiropoulou CF, Morzunov S, Rollin PE, Ksiazek TG, Feldmann H, et al. Genetic identification of a hantavirus associated with an outbreak of acute respiratory illness. Science 1993;262:914-7.
12. Childs JE, Ksiazek TG, Spiropoulou CF, Krebs JW, Morzunov S, Maupin GO, et al. Serologic and genetic identification of ***Peromyscus maniculatus*** as the primary rodent reservoir for a new hantavirus in the southwestern United States. J Infect Dis 1994;169:1271-80.
13. Henttonen H, Vapalahti O, Vaheri A. How many kinds of hantaviruses? Trends in Ecology and Evolution 1996;11:7-8.
14. Otteson EW, Riolo J, Rowe JE, Nichol ST, Ksiazek TG, Rollin PE, et al. Occurrence of hantavirus within the rodent population of northeastern California and Nevada. Am J Trop Med Hyg 1996;54:127-33.
15. Levis S, Rowe JE, Morzunov S, Enria DA, St. Jeor SC. New hantaviruses causing hantavirus pulmonary syndrome in central Argentina. Lancet 1997;349:998-9.
16. Mills JN, Ksiazek TG, Ellis BA, Rollin PE, Nichol ST, Yates TL, et al. Patterns of association with host and habitat: antibody reactive with Sin Nombre virus in small mammals in the major biotic communities of the southwestern United States. Am J Trop Med Hyg 1997;56:273-84.
17. Boone JD, Otteson EW, Villard P, McGwire KC, Rowe JE, St. Jeor SC. Ecology and demography of hantavirus infections in rodent populations in the Walker River Basin of Nevada and California. Am J Trop Med Hyg 1998;59:445-51.
18. Calisher CH, Sweeney W, Mills JN, Beaty BJ. Natural history of Sin Nombre virus in western Colorado. Emerg Infect Dis 1999:5:126-34.
19. McKelvey KS, Noon BR. Incorporating uncertainties in animal location and map classification into habitat relationships modeling. In: Perspectives on uncertainty in ecological data. Springer Verlag. In press, 1999.
20. Stoms DM, Davis FW, Cogan CB. Sensitivity of wildlife habitat models to uncertainties in GIS data. Photogrammetric Engineering and Remote Sensing 1992;58:843-50.
21. Mills JN, Ksiazek TG, Peters CJ, Childs JE. Long-term studies of hantavirus reservoir populations in the southwestern United States: a synthesis. Emerg Infect Dis 1999;5:135-42.
22. Engelthaler DM, Levy CE, Fink TM, Tanda D, Davis T. Short report: decrease in seroprevalence of antibodies to hantavirus in rodents from 1993-1994 hantavirus pulmonary syndrome case sites. Am J Trop Med Hyg 1998;58:737-8.
23. Abbott KD, Ksiazek TG, Mills JN. Long-term hantavirus persistence in rodent populations in central Arizona. Emerg Infect Dis 1999;5:102-12.
24. Billings WD. Vegetational zonation in the Great Basin of western North America. International Union of Biological Sciences. Series B 1951;9:101-22.
25. Scott J, Davis F, Csuti B, Noss R, Butterfield B, Groves C, et al. Gap analysis: a geographical approach to protection of biological diversity. Wildlife Monographs 1993;123:141.
26. Tucker C. Red and photographic infrared linear combinations for monitoring vegetation. Remote Sensing of Environment 1979;8:127-50.
27. Tucker C, Van Praet C, Boerwinkel E, Gaston A. Satellite remote sensing of total dry matter production in the Senegalese Sahel. Remote Sensing of Environment 1983;13:461-74.
28. Wessels K, Van Jaarsveld A, Grimbeek J, Van Der Linde M. An evaluation of the gradsect biological survey method. Biodiversity and Conservation 1998;7:1093.
29. Desert Research Institute. Modeling the spatial and temporal dynamics of hantavirus infection in host populations. Available from URL:http://dia.dri.edu/hanta/

30. Dobson AP. Introduction. In: Grenfell BT, Dobson AP, editors. Ecology of infectious diseases in natural populations. Cambridge: Cambridge University Press; 1995. p. 1-19.
31. Dobson AP, Hudson PJ. Microparasites: observed patterns. In: Grenfell BT, Dobson AP, editors. Ecology of infectious diseases in natural populations. Cambridge: Cambridge University Press; 1995. p. 52-89.
32. Hurlbert SH. Pseudoreplication and the design of ecological field experiments. Ecological Monographs 1984;54:187-211.
33. Tabachnik BG, Fidell LS. Using multivariate statistics. 3rd ed. New York: Harper Collins; 1996. p. 507-8 and 514.
34. Clark L, Pregibon D. Tree-based models. In: Chambers JM, Hastie TJ, editors. Statistical models in S. Pacific Grove (CA): Wadsworth and Brooks/Cole; 1992. p. 377-419.
35. Schumaker NH. Using landscape indices to predict habitat connectivity. Ecology 1996;77:1218-25.
